# The Role of Open Necrosectomy in the Current Management of Acute Necrotizing Pancreatitis: A Review Article

**DOI:** 10.1155/2013/579435

**Published:** 2013-01-28

**Authors:** K. Vasiliadis, C. Papavasiliou, A. Al Nimer, N. Lamprou, C. Makridis

**Affiliations:** First Department of General Surgery, Papageorgiou Hospital, Nea Efkarpia, 564 03 Thessaloniki, Greece

## Abstract

The optimal management of necrotizing pancreatitis continues to evolve. Currently, conservative intensive care treatment represents the primary therapy of acute severe necrotizing pancreatitis, aiming at prevention of organ failure. Following this mode of treatment most patients with sterile necroses can be managed successfully. Surgery might be considered as an option in the late phase of the disease for patients with proven infected pancreatic necroses and organ failure. For these patients surgical debridement is still considered the treatment of choice. However, even for this subgroup of patients, the concept of operative strategy has been recently challenged. Nowadays, it is generally accepted that necrotizing pancreatitis with proven infected necroses as well as septic complications directly caused by pancreatic infection are strong indications for surgical management. However, the question of the most appropriate surgical technique for the treatment of pancreatic necroses remains unsettled. At the same time, recent advances in radiological imaging, new developments in interventional radiology, and other minimal access interventions have revolutionised the management of necrotizing pancreatitis. In light of these controversies, the present paper will focus on the current role of surgery in terms of open necrosectomy in the management of severe acute necrotizing pancreatitis.

## 1. Introduction

Despite the considerable progress in the knowledge of the natural course and pathophysiology of acute pancreatitis (AP), the underlying pathogenetic mechanisms acting during the course of the disease, leading to acinus cell necroses and propagation of necrotizing inflammation, are still to a large extent undefined [1–4]. In the majority of cases, AP comprises clinically a mild transitory form of oedematous-interstitial inflammation, which is self-limiting and resolves spontaneously. However, 15%–20% of patients with AP will develop the more severe form of the disease [5]. Such patients will initially exhibit a hypovolemic state or even shock, followed by fluid sequestration into the pancreas, peripancreatic areas, and intra- and extraperitoneal spaces [6]. The subsequent systemic hyperinflammation may lead to organ dysfunction and/or local complications, such as pancreatic necrosis (PN) and formation of intra-abdominal abscesses and/or pseudocysts [7, 8]. 

Primary therapy of severe necrotizing pancreatitis (SNP) consists of a conservative intensive care treatment, aiming at fluid replacement, sufficient analgesia, and prevention of organ failure. Clinical recommendations call for SNP to be treated in specialized units with multidisciplinary expertise available on site, including intensive care (IC) specialists, interventional endoscopists, diagnostic and interventional radiologists, and surgeons [9–12]. Following such a treatment protocol, most patients with sterile PN can be managed successfully conservatively [13–15]. Although treatment of SNP should be conservative in its early phase, surgery might be considered as an option in the disease's later phase. In fact, surgical debridement is still considered the treatment of choice in patients with proven infected PN, given that they present with organ failure [16]. However, even for this subgroup of patients, the concept of operative strategy has been recently challenged. 

Nowadays, it is generally accepted that SNP with proven infected necrosis as well as septic complications directly caused by pancreatic infection are strong indications for surgical management [17, 18]. But even when the decision to operate is made, the question of the most appropriate surgical technique for the treatment of PN remains unsettled. At the same time, recent advances in radiological imaging, new developments in interventional radiology and other minimal access interventions have revolutionised the management of SNP. 

In light of these controversies, the present paper will focus on the current role of surgery in terms of open necrosectomy in the management of severe acute necrotizing pancreatitis.

## 2. Infected Pancreatic Necrosis

Today, more patients survive the first phase of SNP owing to improvements in IC medicine, thereby increasing the risk of infection of PN and later sepsis [30, 31]. Secondary infection of PN develops in 40%–70% of patients with a mortality rate greater than 20% and is found in 80% of patients dying from AP [32]. In contrast, mortality for sterile PN is low and can be successfully treated by a conservative approach, although surgery might be required for late complications or persistent SNP [11]. 

Isolated pathogens from infected PN commonly include enteric Gram-negative organisms, with *Escherichia coli* predominating (25–35%), followed by Gram-positive organisms (20%), and anaerobes (10–15%). Fungal infection of PN is usually a late event in the disease course and is related to the prolonged use of antibiotic therapy [33]. 

Differentiation between sterile and infected PN is essential for the management of AP. Diagnosis of infected PN can be established by direct CT evidence of retroperitoneal gas or positive cultures of necrotic fine needle aspirates (FNA) [34, 35]. FNA for bacteriology represents a well established method for the identification of infected PN and is indicated in patients with CT-proven necrosis and clinical signs of sepsis [36]. Although some selected cases of AP with positive FNA can be treated without surgery, conservative management of patients with infected PN and multiple organ failure (MOF) is associated with mortality rates of up to 100% [37]. In contrast, the mortality rate for patients with infected PN could be decreased to approximately 20% following surgical treatment in specialised centers [24, 25]. There is general consensus that proven infected PN as well as septic complications resulting from pancreatic infection are well established indications for surgical treatment (Tables 1 and 2).

## 3. The Role of Surgery in the Management of Acute Necrotizing Pancreatitis

More than 110 years have passed since Fitz [38] first described AP in his classic paper. At the time, surgical therapy was indicated as a desperate attempt to reverse the disease's lethal course and lower its dramatically high mortality rates. 

Following the introduction of serum amylase assays as a reliable parameter for the diagnosis of the disease in 1925, it became evident that the majority of patients with AP had a mild transitory form of the disease that was self-limiting and resolved spontaneously. With that knowledge, the therapeutic approach was directed toward conservative management [39–41]. Nevertheless, the outcome of patients with the severe form of the disease remained poor, forcing the medical community to reassess the role of surgical intervention in this specific subset of patients [42, 43]. As a consequence, a variety of surgical approaches were implemented ranging from minor procedures, such as pancreatic drainage, to aggressive interventions, such as total pancreatectomy, with variable but generally disappointing results [44–50]. 

In the following years, new advances in radiological imaging pointed to an association between the incidence and severity of complications of AP and the presence of PN [51, 52]. Several factors have been implicated as major determinants for the poor outcome, including the extent of intra- and extrapancreatic necrosis [53], the infection of PN [54], and recently, early onset and persistent multiple organ dysfunction syndrome (MODS) [19, 55–58]. 

In the past 25 years, a multidisciplinary therapeutic approach of IC management combined with surgical necrosectomy and drainage of the peripancreatic spaces decreased the mortality of SNP to nearly 20% [1–4]. Two additional major therapeutic concepts in the context of surgical therapy of PN were introduced in the 1980s and gained general acceptance because they offered further evacuation of necrotic or infected pancreatic and peripancreatic tissue. These were the open approach by controlled packing or repeated, planned reoperative debridement [20, 59, 60], and the closed approach with continuous lavage [28, 61–65] or simple drainage [66–68]. 

Despite the benefits of these new surgical concepts, postoperative morbidity and procedure-related complications still remained high, thereby posing a therapeutic dilemma for the management of patients with SNP [69, 70]. Considering the prognostic importance of infection of PN, a completely conservative approach was attempted during the early 1990s for patients with sterile PN. In this patient group the mortality rate was favorable if secondary infection was prevented. Thus, since 1991 when Bradley III and Allen [71] introduced the concept of conservative treatment in noninfected PN, the therapeutic approach of patients with PN once again shifted away from operative toward conservative approaches [72–74], changing the indications and timing of surgical intervention substantially. Additionally, several diagnostic and therapeutic protocols, such as guided fine-needle aspiration of necrosis [75], early endoscopic retrograde cholangiopancreatography (ERCP) in patients with acute biliary pancreatitis [75, 76], prophylactic antibiotics [77], and early enteral feeding helped to correctly diagnose and even decrease the occurrence of subsequent complications in this subset of patients. 

The most recent International Association of Pancreatology Guidelines recommend that a patient with infected PN has to undergo surgery in the third or fourth week after onset of symptoms [11]. However, it should be noted that postponing surgical intervention in PN can lead to prolonged use of antibiotics and an increased antibiotic resistance and higher incidence of Candida infection [78, 79]. Besselink and coworkers [80] in their study strongly advised against surgical intervention in the first 14 days even in the presence of MOF, and urged postponing necrosectomy until day 30 [80]. Currently, the overall percentage of patients with SNP ultimately subjected to operative treatment has decreased to less than 20%.

## 4. Rationale and Indications of Necrosectomy

The concept of necrosectomy performed with rigorous preservation of vital pancreatic tissue can berationalized by suggesting that it accomplishes locally focused control of necrosis and pancreatogenic ascites from the lesser sac and the peritoneal cavity, which in turn interrupt the devastating progress of inflammation and the systemic release of various inflammatory mediators that account for remote organ failure [81, 82]. In addition, lavage of the lesser sac and peritoneal cavity represents a useful adjunct of necrosectomy and is supported by the results of several randomized controlled trials [21, 83, 84]. Necrosectomy should be restricted in patients with PN in whom conservative or interventional treatment has failed. The initial treatment should include maximum IC management continued for at least 2 weeks after symptom onset. Although the development of MODS and MOF in this subset of patients can frequently complicate the early phase of the clinical course of the disease, it should be noted that at least 50% of these patients respond well to IC treatment [85, 86]. Therefore, prolonged IC treatment is essential for the selection of patients who do not require surgery. The usefulness of a conservative management concept in patients with PN is further supported by the clinical observation that the effectiveness of necrosectomy is directly related to the grade of demarcation of necrosis, which develops at the end of the 2nd week after symptom onset. The presence of documented infection of PN is nowadays a uniformly accepted indication for interventional drainage. However, this indication for surgery has also been challenged [74]. Based on recent data, one can conclude that extended conservative treatment protocols may result in a favorable outcome. Nonetheless, a clear association between infected necrosis and septic MOF, the ultimate cause of death, has been well established [87]. It therefore remains to be proven how much conservative treatment a critically ill patient with infected necrosis can bear before systemic sepsis becomes uncontrolled and a point of no return is reached. 

## 5. Timing of Necrosectomy

The clinical course of patients with SNP can progress to a critical condition within a few hours or days after the onset of symptoms. In the past, when MODS or MOF were complicating the clinical course of the disease, early surgical intervention was favoured. However, its beneficial effect on patients' outcome was rather disappointing, as it was associated with mortality rates of up to 65% [47, 88]. In the only prospective randomised trial comparing early (within 72 hours of symptoms) with late (at least 12 days after onset) pancreatic resection/debridement in patients with SNP, mortality rates were 56% and 27%, respectively [88]. This trial had to be terminated because of concern about the very high mortality of early surgery. In a recent retrospective study, Besselink et al. [80] strongly advised avoidance of surgical intervention in the first 14 days even in the presence of MOF, and withholding of necrosectomy until day 30. Today, there is general agreement that surgery in SNP should be performed as late as possible [11]. The third to fourth week after the onset of the disease is agreed as providing optimal operative conditions with well demarcated necrotic tissue present, thus limiting the extent of surgery to pure debridement and to only one single intervention. This approach decreases the risk of bleeding, minimises the surgery related loss of vital tissue, and thus reduces the rate of endocrine and exocrine pancreatic insufficiency. Only in the case of proven infected necrosis or in the presence of rare complications, such as massive bleeding or bowel perforation, must early surgery be performed [9, 11]. 

## 6. Open Pancreatic Necrosectomy/Debridement

During the past decades a wide spectrum of surgical procedures has been advocated for the surgical management of SNP, ranging from nonresecting procedures to major, aggressive, extensively resecting operations. Nevertheless, neither “conservative” surgical strategies [89], nor aggressive resections [44–49], have accomplished a significant reduction in the overall mortality of SNP. The reason for such disappointing results is likely linked to the fact that none of these treatment protocols sufficiently addressed the underlining pathophysiological mechanisms of the disease. Specifically, some “conservative” procedures neither approached the retroperitoneal spaces, nor evacuated infected necroses, while major resectional operations, aiming at the radical removal of the gland, exposed the already severely ill patients to additional stress by removing still viable pancreatic parenchyma and neighboring organs. Clearly, the appropriate operative technique for the treatment of SNP should consist of careful removal of PN combined with rigorus preservation of vital pancreatic tissue and neighboring organs. 

This new concept in the operative techniques resulted in a significant reduction in mortality rates, that were originally greater than 50%, to about 20% [66]. However, despite these improvements in patients' postoperative outcome, recurrent post-necrosectomy intra-abdominal sepsis, because of inadequate drainage or incomplete necrosectomy, continues to pose a major drawback [19]. Currently, the aim of operative necrosectomy is to remove the focus of necrotic and infected tissue so that further complications are avoided by blocking the inflammatory process and stopping the release of proinflammatory mediators. 

Today, the generally agreed upon principles of necrosectomy are minimization of injury to viable tissues and maximization of postoperative removal of exudative fluid and extravasated pancreatic exocrine secretions from the operative bed [9, 90].

The four principal, surgical techniques currently performed to evacuate necrotic or infected pancreatic and peripancreatic tissue are:

- necrosectomy combined with open packing [91];
- planned staged relaparotomies with repeated lavage [23];
- closed continuous lavage of the lesser sac and retroperitoneum [27];
- closed packing [25].

Necrosectomy is traditionally performed via an open route. Adequate debridement is usually achieved with a single operation. A longitudinal midline incision allows the assessment of the entire abdominal cavity, irrigation of the entire abdomen, and the performance of a diverting ileostomy provided that the necrotic process involves the retrocolic area. After the abdominal cavity is entered, the gastrocolic and the duodenocolic ligaments are divided close to the greater curvature of the stomach, and the pancreas is exposed. Once the focus of necrosis is exposed, debridement is carried out bluntly. Open necrosectomy avoids removal of vital tissue and reduces bleeding complications. After all loose debris has been removed the retroperitoneal cavity is irrigated with several litres of normal saline solution. As a general comment it should be noted that although necrosectomy is performed in a more or less identical fashion, the four techniques differ in the way they provide exit channels for further slough and infected debris. Series of patients treated with open necrosectomy atexperienced care centersshowed mortality rates below 15% for all four techniques [92].

## 7. Technique of Necrosectomy Combined with Open Packing

The open packing technique is based on the continuous reoperation principle, with open lavage of the necrotic areas. Manual exploration and visual inspection of the entire peritoneal cavity is performed trough a left subcostal incision to determine the extent of necroses. Necrotic spaces are unroofed in a controlled fashion. Depending on the extent of necrosis, the incision can be extended to perform extensive unroofing of the retroperitoneum and sequestrectomy. After debridement, the lesser sac is lined with a ring of non-adherent material to protect adjacent intestinal surfaces and to prevent injuries, and packed. A needle catheter jejunostomy for eventual enteral feeding is also inserted. The abdomen is left open and the patient is returned to the operating room every 24 hrs to 48 hrs for further debridement and repacking until no further necrosis is evident and healthy granulations appear. Then, the abdominal wound is permitted to heal entirely by secondary intention or it can be closed over drains placed in the area of debridement, with or without lavage of the cavity [91, 93]. 

## 8. Technique of Planned Staged Relaparotomies with Repeated Lavage

After entering the peritoneal cavity via a midline vertical incision the following take place: a systematic manual exploration and visual inspection of the entire pancreas as well as an exploration to determine the extent of necrosis in both paracolic gutters, the root of the small bowel mesentery below the transverse mesocolon, and the suprapancreatic retroperitoneal tissues. Entrance into the lesser omental sac is accomplished through the gastrocolic ligament which is probed manually to identify the cavity containing the necrosis in the lesser sac. The necrotic space is unroofed in a controlled fashion, with care to protect neighboring vital anatomical structures. Necrosectomy is carried out by a gentle, manual, blunt, “scooping out” dissection. According to this technique, all devitalized tissues amenable to debridement are removed at the initial necrosectomy without inducing hemorrhage. After necrosectomy, an extensive irrigation of the debrided areas is performed and abdominal wall closure proceeds with a zipper sewn to the fascia. Reoperation is performed 48 h after the initial procedure and uses the same technique. The zipper is opened and the abdomen is fully reexplored in a systematic fashion. Additional necrosectomy and blunt debridement are performed as needed. The process is repeated at 48 hrs intervals until the necrotizing process has been arrested. If at the initial operation a complete necrosectomy is performed, then the abdomen is closed without planned reexploration. When all necrotic debris has been removed, the abdomen is closed over drains. Drains are routed below the liver and posterior to the hepatic flexure on the right side and posterior to the splenic flexure inferior to the lower pole of the spleen on the left side. A gastrostomy tube for gastric decompression and a needle catheter jejunostomy for eventual enteral feeding are inserted. At final closure, the zipper is removed, and the abdominal wall is closed with nonabsorbable suture. The skin remains packed open [23, 94].

## 9. Technique of Closed Continuous Lavage of the Lesser Sac and Retroperitoneum

After entering the peritoneal cavity trough a midline vertical incision, the lesser sac is opened by dividing the duodenocolic and gastrocolic ligaments close to the greater curvature of the stomach inferior to the gastroepiploic vessels. All identified fluid collections are opened and evacuated by suction. Debridement of necrotic pancreatic and peripancreatic fatty tissue is accomplished mainly by blunt digital dissection. Following debridement in the lesser sac, necrotic tissue is systematically sought in the retroperitoneum behind the transverse, ascending, and descending colon, and down to Gerota's fascia, and all foci of necrosis are removed by blunt dissection. After necrosectomy, the pancreatic area and the retroperitoneal cavity are irrigated generously with normal saline. Bleeding from the pancreas is controlled by transfixion sutures using nonabsorbable, monofilament suture material. After irrigation and hemostasis, four drainage catheters (two double-lumen Salem type sump tubes (20–24 French) and two single-lumen silicone rubber tubes (28 to 32 French)), two from each side, are directed to the contralateral side of the peripancreatic space and placed with the tip of the catheter at the head and tail of the pancreas behind the ascending and descending colon. The smaller lumen of the Salem drains is used for the inflow of the lavage and the larger lumen for the outflow. Following the placement of drains, the gastrocolic and duodenocolic ligaments are resutured together to create a closed peripancreatic compartment to allow for contained postoperative lavage of the lesser sac and involved retroperitoneum. In cases of marked meso- and retrocolic necrosis threatening the viability of the transverse colon, a diverting ileostomy is created in the right lower quadrant as the last step of the procedure. Thirty-five to forty litres of lavage fluid (standard peritoneal dialysis fluid) are used in the first days. Lavage volume can be reduced depending on the appearance of the effluent and the clinical course. Drains can be removed within the next 2 to 3 weeks [27, 95]. 

## 10. Technique of Closed Packing

The goal of this technique is to perform a single operation with thorough debridement and removal of necrotic and infected tissue, while minimizing the need for reoperation or subsequent pancreatic drainage. Usually, the peritoneal cavity is entered trough a midline vertical incision that allows for better exposure and optimal placement of drains. The transverse colon is elevated anteriorly and access to the lesser sac is gained via the left mesocolon. When necrosis is extensive, there is often bulging of the necrotic process at the site of left mesocolon. In such situations, entry into the necrotic cavity is made bluntly with a clamp or the surgeon's finger. The opening is then enlarged, and the cavity is explored using two fingers. All fluid accumulations are evacuated and sent for culture. In cases of extension of the necrotic process to the right of the lesser sac, an additional incision in the right mesocolon is made and, if necessary, the middle colic vessels are clamped, suture ligated, and divided. Necrosectomy is done bluntly with fingers or with a sponge. All the recesses of the cavity are broken, to remove the debris and necrotic material. During necrosectomy, firm attachments are either clamped and tied or left alone. When necrosis extends deep into the perirenal spaces and cannot be accessed through the mesocolon, the respective paracolic gutters are opened to remove all necrotic tissue. After completing the necrosectomy/debridement, the pancreatic bed is irrigated with several liters of normal saline. Following this, stuffed Penrose drains (“cigarette drains”, made using 3/4-inch Penrose drains stuffed with 2 gauze sponges) are used for packing the large, stiff cavity that results after debridement. These drains are introduced in the abdomen through separate stab wounds to the side of the midline incision. Such drain fills the cavity and provides compression, instead of strictly draining the area. The number of drains depends on the size of the cavity. In addition, soft, round, closed suction silicone Jackson-Pratt drains are introduced in each major locale of the debridement cavity. The stuffed Penrose drains are removed 5–7 days postoperatively. The Penrose drains are removed singly every other day, which allows the cavity to close gradually. The closed suction silicon drains are removed last, when they have no more output. A gastrostomy tube is also placed at the time of the debridement in the majority of patients. It prevents the need for a nasogastric tube and can be used eventually for enteric feeding. In patients with cholecystitis, a cholecystectomy is performed provided the degree of inflammation in the right upper quadrant makes it safe. Finally the abdomen is closed, primarily in routine fashion [25, 96].

## 11. Benefits and Limitations of Open Necrosectomy Techniques

“Open packing” and “planned staged relaparotomies with repeated lavage” are associated with a significant decrease in the incidence of recurrent post-necrosectomy intra-abdominal sepsis when compared to single necrosectomy. However, both of these methods require repeated laparotomies and intra-abdominal manipulations before final abdominal closure, which results in a significantly higher incidence of postoperative complications. There is a positive correlation between surgical reinterventions and high incidence of small and large bowel fistulas, pancreatic fistulas, gastric outlet obstruction syndrome, incisional hernias, and hemorrhage from the cavity of repeated debridement, which may suggest an iatrogenic etiology. Specifically, the number of pancreatic and colonic fistulas is significantly higher for these two methods when compared to necrosectomy with subsequent cleavage of the necrotic debris by “closed continuous lavage” or “closed packing” [13, 22, 23, 25, 93]. Such data restrict the general application of this treatment protocol, which should probably be considered in cases when early debridement is indicated [11]. 

The main difference between the “open packing” and “planned staged relaparotomies with repeated lavage” necrosectomy techniques, and “closed continuous lavage” and “closed packing”, techniques is that the latter two approaches require a postoperative method to continuously evacuate both fluid and necrotic debris [11]. As a consequence, when evacuation of debris and inflammatory fluids is successful, reoperations are rarely necessary. Thus, postoperative morbidity, especially the incidence of gastrointestinal fistulas and incisional hernias, is reduced (Tables 3 and 4). 

The “closed continuous lavage” and “closed packing” approaches are associated with similar incidences in postoperative morbidity, relaparotomies, and mortality. Currently, “necrosectomy and subsequent closed continuous lavage of the lesser sac” is the most commonly applied open approach [9, 11]. 

The reported differing success rates among similar surgical approaches toward PN underscore the difficulties in comparing these techniques (Tables 3 and 4). The majority of these surgical approaches are associated with an average mortality rate between 10% and 20%, which is significantly higher in patients with established MOF [20]. Thus, in the absence of randomized controlled trials, it is impossible to determine the hidden effects of factors, such as referral pattern, patient selection, comorbidity of patients, presurgical percutaneous management, and indication for surgery within the literature. Besides, it is extremely difficult and ethically challenging to design a randomized trial on when and how to operate, or even when not to operate critically ill patients with SNP. 

Long-term outcomes data concerning recovery and pancreatic function after open necrosectomy are scarce. Most available data refer to function after resection, which is accompanied by a high incidence of diabetes. It has to be expected that about two-thirds of debrided patients will develop exocrine and endocrine insufficiency [97]. 

The persistent high mortality rates among operated patients with infected PN have forced the medical community to develop and implement several minimal invasive techniques including radiological, endoscopic, and minimal invasive interventions as alternative therapeutic approaches [9]. Proponents of using minimally invasive technologies in this clinical setting cite an intent to minimize the physiological insult to patients who are already critically ill and claim that the role of open necrosectomy should be re-evaluated, and that less aggressive treatments integrated in a multidisciplinary approach can reduce the morbidity and mortality rates that are associated with open necrosectomy [98, 99]. 

In addition, a recent report by van Santvoort et al. [29] has argued against the need of infected PN patients to undergo open necrosectomy. This multicenter randomized controlled study evaluated a step-up approach for the treatment of infected pancreatic necrosis, utilizing endoscopic and percutaneous techniques, and minimally invasive necrosectomy with a retroperitoneal approach in the event of ineffective endoscopic and percutaneous techniques. This study compared the step-up approach to the standard open necrosectomy. It was demonstrated that when using such an approach, the frequency of major complications such as organ failure, perforation, fistula or even death was significantly lower than in those patients who received conventional open necrosectomy. In the long-term, development of diabetes was also less frequent in those receiving less aggressive therapy. These findings, in combination with data from several similar series [100–102] have challenged the surgical axiom that open necrosectomy is mandatory for all patients with infected PN. 

However, the study of van Santvoort et al. [29] has certain limitations, including the narrow applicability of the step-up approach, because of the need for a retroperitoneal access route, the need in both groups for multiple operative interventions, and a higher overall mortality than observed in open necrosectomy as reported in recent studies [22, 26]. In addition, this study addresses a comparison of percutaneous drainage plus video-assisted retroperitoneal debridement (VARD) with open necrosectomy and does not address whether VARD is superior to open necrosectomy when necrosectomy is needed [103]. Furthermore, it should be stressed that safe retroperitoneal access and necrosectomy is possible in some, but not all, patients depending on the size and localization of infected necrosis while the vast majority of studies on minimally invasive surgery have small patient sample size, are analysed retrospectively, and involve selected patients with an enormous variation of comorbidities and disease severity [9]. 

Regardless of the possible limitations of studies dealing with minimally invasive approaches, it should be noted that they definitively contribute to an important synthesis and integration of evolving techniques [103]. Considering that these alternative treatment options still require both careful judgment and special skills, one should be very cautious in the application of new technologies, in the absence of well-designed clinical trials.

## 12. Conclusion

While the debate and controversies on treatmentoptions for the optimalmanagement of SNP, are still ongoing, open surgical debridement remains the “gold standard” for the treatment of infected pancreatic and peripancreatic necrosis. “Necrosectomy and subsequent closed continuous lavage of the lesser sac” is, among the open necrosectomy techniques, the one with the lowest morbidity.

## Figures and Tables

**Table 1:** Indications for open necrosectomy [14, 17].

The operation should be undertaken as late as possible, when necroses have been ceased, viable and nonviable tissues are well demarcated, and infected necrotic tissues are “walled off”.	
Pancreatic and/or peripancreatic necrosis complicated by documented infection (guided FNA culture or extraluminal retroperitoneal gas)	
Sterile necrosis	
(a) Progressive clinical deterioration despite maximal medical treatment	
(b) “Fulminant acute pancreatitis”	
Massive hemorrhage or hollow viscus perforation	

**Table 2:** Contraindications for open necrosectomy [14, 17].

Pancreatic and/or peripancreatic necrosis without evidence of infection or clinical deterioration	
Early operation (within 1 week from onset of acute pancreatitis)	

**Table 3:** Outcome of different techniques for open necrosectomy.

Technique	Patients (*n*)	Patients with infected necrosis (%)	Mortality (%)	Relaparotomy (*n*)
Open packing				
Bradley III, 1993 [19]	71	100	15	1–5/pt.
Bosscha et al., 1998 [20]	28	100	39	17 mean/pt.
Nieuwenhuijs et al., 2003 [21]	38	—	47	—
Howard et al., 2007 [22]	102	75	12	—
Planned relaparotomies				
Sarr et al., 1991 [23]	23	75	17	2–>5/pt.
Tsiotos et al., 1998 [24]	72	79	25	1–7/pt.
Closed packing				
Fernandez-del Castillo et al., 1998 [25]	64	56	6	11 (17%)
Rodriguez et al., 2008 [26]	167	68	11	14 (11%)
Closed continuous lavage				
Beger et al., 1988 [27]	95	37	8	26 (27%)
Farkas et al., 2006 [28]	123	100	7	—
Büchler et al., 2000 [13]	29	27	24	6 (22%)
Nieuwenhuijs et al., 2003 [21]	21	—	33	—
van Santvoort et al., 2010 [29]	45	93	16	1–7/pt.

**Table 4:** Complications of different techniques for open necrosectomy.

Technique	Patients (*n*)	Fistulas (pancreatic/enteric)	Haemorrhage (%)
Open packing			
Bradley III, 1993 [19]	71	46%	7%
Bosscha et al., 1998 [20]	28	25%	50%
Howard et al., 2007 [22]	102	54%	4%
Planned relaparotomies			
Sarr et al., 1991 [23]	23	(26%/52%)	26%
Tsiotos et al., 1998 [24]	72	(19%/27%)	18%
Closed packing			
Fernandez-del Castillo et al., 1998 [25]	64	(53%/16%)	3%
Rodriguez et al., 2008 [26]	167	(36%/14%)	4%
Closed continuous lavage			
Farkas et al., 2006 [28]	123	(13%/1%)	2%
van Santvoort et al., 2010 [29]	45	(38%/22%)	22%
